# Quantitative digital histopathology and machine learning to predict pathological complete response to chemotherapy in breast cancer patients using pre-treatment tumor biopsies

**DOI:** 10.1038/s41598-022-13917-4

**Published:** 2022-06-11

**Authors:** Khadijeh Saednia, Andrew Lagree, Marie A. Alera, Lauren Fleshner, Audrey Shiner, Ethan Law, Brianna Law, David W. Dodington, Fang-I Lu, William T. Tran, Ali Sadeghi-Naini

**Affiliations:** 1 Department of Electrical Engineering and Computer Science, Lassonde School of Engineering, York University, Toronto, ON Canada; 2 Department of Radiation Oncology, Sunnybrook Health Sciences Center, Toronto, ON Canada; 3 Department of Laboratory Medicine and Pathobiology, University of Toronto, Toronto, ON Canada; 4 Department of Radiation Oncology, University of Toronto, Toronto, ON Canada; 5 Temerity Centre for AI Research and Education in Medicine, University of Toronto, Toronto, ON Canada; 6 Physical Sciences Platform, Sunnybrook Research Institute, Toronto, ON Canada

**Keywords:** Predictive markers, Prognostic markers, Biomedical engineering, Breast cancer, Cancer

## Abstract

Complete pathological response (pCR) to neoadjuvant chemotherapy (NAC) is a prognostic factor for breast cancer (BC) patients and is correlated with improved survival. However, pCR rates are variable to standard NAC, depending on BC subtype. This study investigates quantitative digital histopathology coupled with machine learning (ML) to predict NAC response a priori. Clinicopathologic data and digitized slides of BC core needle biopsies were collected from 149 patients treated with NAC. The nuclei within the tumor regions were segmented on the histology images of biopsy samples using a weighted U-Net model. Five pathomic feature subsets were extracted from segmented digitized samples, including the morphological, intensity-based, texture, graph-based and wavelet features. Seven ML experiments were conducted with different feature sets to develop a prediction model of therapy response using a gradient boosting machine with decision trees. The models were trained and optimized using a five-fold cross validation on the training data and evaluated using an unseen independent test set. The prediction model developed with the best clinical features (tumor size, tumor grade, age, and ER, PR, HER2 status) demonstrated an area under the ROC curve (AUC) of 0.73. Various pathomic feature subsets resulted in models with AUCs in the range of 0.67 and 0.87, with the best results associated with the graph-based and wavelet features. The selected features among all subsets of the pathomic and clinicopathologic features included four wavelet and three graph-based features and no clinical features. The predictive model developed with these features outperformed the other models, with an AUC of 0.90, a sensitivity of 85% and a specificity of 82% on the independent test set. The results demonstrated the potential of quantitative digital histopathology features integrated with ML methods in predicting BC response to NAC. This study is a step forward towards precision oncology for BC patients to potentially guide future therapies.

## Introduction

Breast cancer (BC) is the most prevalent cancer diagnosed among women, and the second cause of cancer-related death worldwide^1^. The annual rate of BC occurrence has increased by 0.3% in the United States^2^, and associated with a risk of one in eight women who will develop BC^1^. Approximately 5–20% of diagnoses include locally advanced breast cancer (LABC)^3^, defined as stage $\mathrm{\rm I}\mathrm{\rm I}\mathrm{\rm I}$ and a subgroup of stage $\mathrm{\rm I}\mathrm{\rm I}$B BC^4^. It typically comprises tumors larger than 5 cm, may involve skin or chest wall invasion, or with extensive axillary lymph node metastases^4,5^. Due to the high risk of cancer progression, metastatic spread, and loco-regional recurrence, LABC is associated with a poorer prognosis compared to early-stage BC^3–5^. The 10-year survival is approximately 44%, which is dependent on BC subtype and response to therapies^6^. Definitive treatment for LABC includes neoadjuvant chemotherapy (NAC) followed by surgery^7^. However, only 10–30% of LABC patients demonstrate pathological complete response (pCR) to NAC, defined as a complete clearance of invasive carcinoma in the breast and regional lymph nodes^8–11^. Previous studies have shown a correlation between pCR and improved 5-year survival of up to 70%^12–15^. However, pathologic assessment is carried out after surgery, which limits the opportunity to adapt NAC treatments according to tumor response. Accordingly, early prediction of treatment response is needed to guide cancer therapy decisions based on individualized patient factors.

Several studies have investigated imaging biomarkers for early diagnosis, prognosis and the prediction of treatment responses in BC^3–5^. However, histological examination remains the standard for cancer diagnosis, while genetic and immunohistochemical assessments may be used for prognosis and treatment outcome prediction^16,17^. Machine learning (ML) algorithms, along with the development of whole slide imaging, has opened new research directions for early assessment of therapy response using quantitative digital histopathology. Digital pathology has the potential to yield large datasets from microscopic imaging and for automated analysis. Availability of such data permits development of computational tools and data-driven ML algorithms to process and interpret different types of tissue in high-resolution histopathology images and derive quantitative features for various diagnostic and prognostic applications^18^. Recent studies have demonstrated promising results for predicting cancer treatment outcome and recurrence using a combination of quantitative imaging (radiomic) and digital histopathology (pathomic) features^19,20^.

The objective of this study is to investigate ML methods coupled with quantitative digital histopathology to predict pCR to neoadjuvant chemotherapy in BC patients using pre-treatment biopsy specimens.

## Materials and methods

### Study protocol and data acquisition

This investigation was a single institution, retrospective study. Ethical approval was obtained from the institutional ethics review board (IRB) at Sunnybrook Health Sciences Centre, Toronto, Canada, prior to data collection and analysis; research was conducted in accordance with the Declaration of Helsinki. As this was a retrospective non-interventional study, a consent waiver was obtained from the IRB under the provision of the Canadian Tri-Council Policy Statement 2 (TCPS; 2018) Articles 3.1–3.5 and 3.7A (i.e., Ethical Conduct for Research Involving Humans). All study data were anonymized; specifically, patient identifiers were removed from each sample prior to analysis. Patients were included in the study based on the following inclusion criteria: confirmed diagnosis of invasive breast cancer*,* age (18 +), and undergoing Anthracycline and/or Taxane based neoadjuvant chemotherapy followed by surgery. There were 149 patients included in the study. All patients had a breast core needle biopsy before NAC with a pathological review as part of their standard of care. Clinicopathological and imaging information were collected for all patients. Clinical data included patient age, menopausal status (pre/post), clinical tumor size (largest radiologically reported dimension from either mammogram ultrasound or magnetic resonance imaging; mm), histological type (ductal versus lobular), Nottingham grade (G1/G2/G3), and the presence or absence of inflammatory cancer (defined as breast carcinoma with dermal lymphatic invasion). Estrogen receptor (ER) status (+/−), progesterone receptor (PR) status (+/−), human epidermal growth factor receptor-2 (HER2) status (+/−) were also obtained for all patients.

Treatment response endpoints were evaluated after surgery and classified into pathological complete response (pCR) versus pathological non-complete response (non-pCR), as ground truth labels for subsequent modelling. A standard assessment method using the residual cancer burden index (RCBI)^21^ was employed for ground truth labeling of response. An RCBI score of 0 (i.e. pCR) was defined as the absence of residual invasive and *nodal* disease^21^. Patients who demonstrated residual disease were classified as non-pCR (i.e., RCBI > 0). All pathology reviews (pre-treatment and post-surgery histopathology) were evaluated by board-certified breast pathologists and as part of the patient’s standard of care. Similarly, radiological reporting was carried out at the time of diagnosis by board-certified breast radiologists. The patients were randomly partitioned into a training (75%; n = 111 patients) and an unseen test set (25%; n = 38 patients). The training set was used for feature reduction/selection and development of predictive models (described below). The test set was used to evaluate the performance of the predictive models independently.

### Core biopsy sample preparation

Formalin-fixed paraffin embedded (FFPE) blocks containing core biopsy specimens obtained from each patient at pre-treatment were microtomed into 4 µm sections. Specimens were prepared onto glass slides and stained with hematoxylin and eosin (H&E). The slides were digitized into whole slide images (WSI) using a TissueScope LE digital pathology image scanner (Huron Digital Pathology Inc, St. Jacobs, Canada) at 40 × magnification. All WSI were reviewed to ensure image integrity before image processing and analysis. If any image was distorted, blurry, or contained occlusions, the associated slide was re-imaged.

### Preprocessing of histology images and pathomic feature extraction

**Figure 1:**
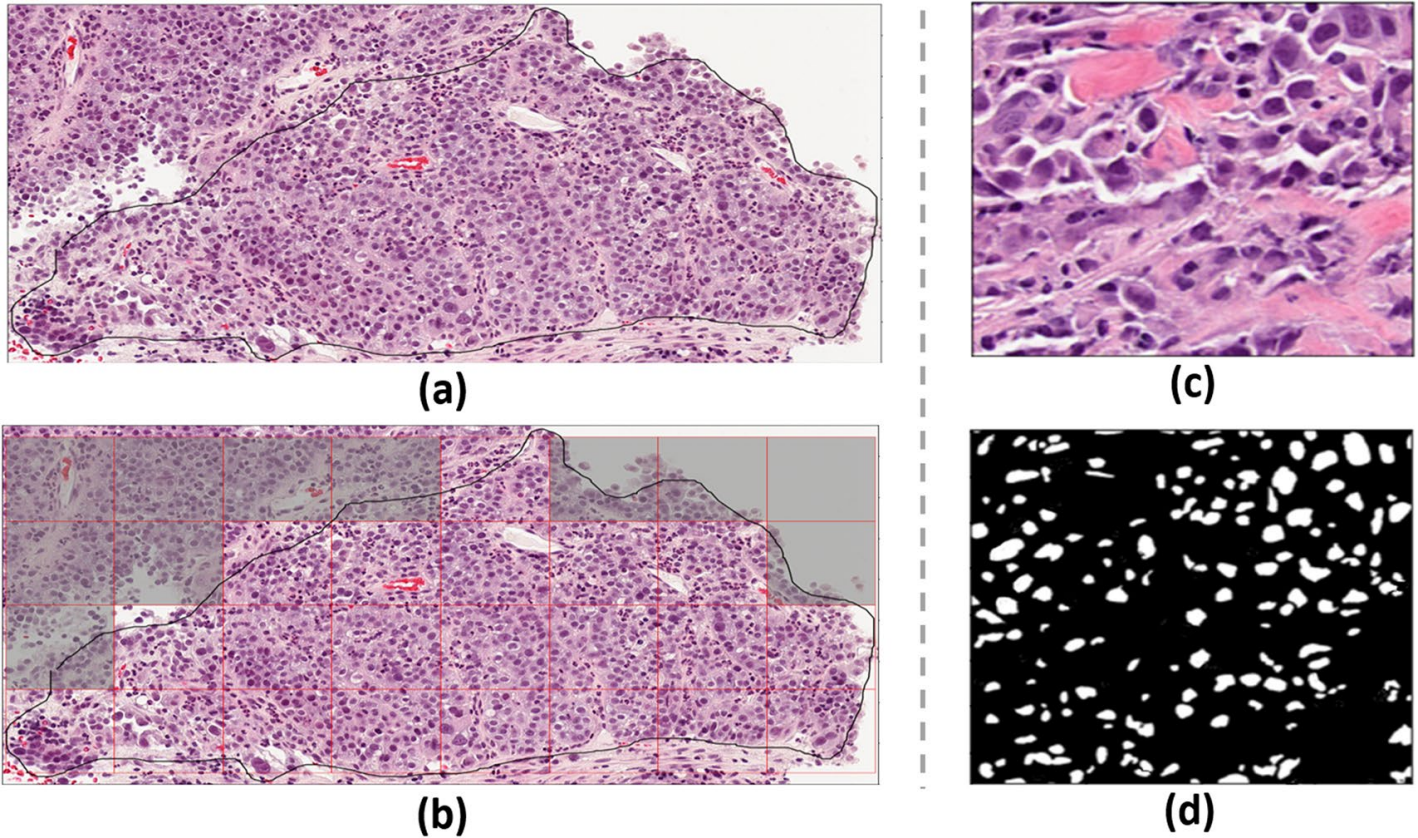
(**a**) An example of tumor bed annotation on a representative segment of the core on WSI (2188 × 4124 pixels), (**b**) the non-overlapping 768 × 768 pixel tiles extracted from the tumor region with the excluded tiles shaded (less than 50% tumor or more than 10% white background), (**c**) an extracted tile with 100% tumor tissue, and (**d**) the generated binary mask of the nuclei in the tile.

The tumor regions were annotated on the WSIs by an expert pathologist using the Sedeen software package^22^. The pre-processing steps were performed on three-channel RGB images. The tumor region annotations were preprocessed to extract non-overlapping tiles with a size of 768 × 768 pixels by including tumor margins, when required. From the extracted tiles only the ones with more than 50% tumor tissue and less than 10% white background were retained for analysis (Fig. 1a,b). A pre-trained weighted U-Net based model was utilized to segment the nuclei in each tile accurately^23^. Histology images from the Cancer Imaging Archive (TCIA) and the Multi-Organ Nucleus Segmentation (MoNuSeg) datasets were used to train the model^24,25^. Each tile was patched to 256 × 256 pixel patches with 128 pixels overlap between the adjacent patches. After segmenting the nuclei, the patches were merged by averaging over the output probability map of the segmentation model within the overlapped regions. The binary nuclei mask for each tile was generated by thresholding the associated averaged probability map with a threshold level of 0.5. The detected nuclei with less than 50 pixels were eliminated in the generated masks based on the empirical observation that the actual nuclei cannot include less than 50 pixels on the histology images acquired at 40 × magnification. Figure 1c,d shows a tile extracted from the tumor region of a representative WSI and the binary nuclei mask generated for it.

Using the HistomicsTK^26^ and PyRadiomics^27^ open-source packages, 549 pathomic features were extracted from each image tile for analysis. The features were representative of five categories: nuclear morphology and Fourier shape descriptors (16 features)^28^, nuclear intensity and gradient features (20 features)^29^, first- and second-order texture features (93 features)^30^, graph-based features (49 features)^31^ and wavelet features consisting of intensity, gradient and texture features extracted from wavelet filtered images (371 features)^32^. The morphological features and Fourier shape descriptors as well as the graph-based features were derived using the binary nuclei masks. The binary masks and the grey-scale image tiles were used to calculate the nuclear intensity, gradient, texture and wavelet features. The extracted features were averaged over all image tiles associated with each WSI to obtain the overall features for each patient. The number of nuclei in each image tile was applied to derive a weighting factor for the tile in calculating the averaged features.

### Feature reduction/selection and tumor response prediction

The clinical and pathomic features were analyzed through a feature reduction and selection process on the training set to develop optimal biomarkers for NAC response prediction. Seven different experiments were conducted to analyze different feature subsets including the clinical, morphological, intensity-based, texture, graph-based and the wavelet feature subsets, in addition to a union of all feature subsets as the initial feature set. All the features were normalized to scale between zero and one before the analysis. A gradient boosting machine (GBM) with decision trees was trained as the classifier for response prediction in each experiment to calculate the contribution of each feature in the associated feature subset to the prediction model based on the importance gain score^33^. The first few features with highest contribution to the model that demonstrated a meaningful difference in the importance gain score compared to rest of the features were selected in each experiment and included in the biomarker.

The GBM model was adapted to develop a predictive model of NAC response using the optimum biomarker in each experiment. A five-fold cross validation on the training set was used with area under the receiver operating characteristic (ROC) curve (AUC) as the criteria to optimize the model hyperparameters. To address the imbalance issue of the dataset, the training samples of the minority class (pCR) in each round were oversampled to a double number using the SMOTE method^34^. The final predictive model was developed using the entire training set with oversampled minority class, a learning rate of 0.1, a maximum depth of 10, and 2000 estimators. The performance of the predictive model with the optimal biomarker was subsequently evaluated on the independent test set using accuracy, sensitivity, specificity, and AUC. A threshold value of 0.5 was used as the cut-off to calculate the sensitivity and specificity.

## Results

Table 1 shows clinical and pathological characteristics of the patients in the training and test sets. Among the 149 patients, 57.7%, 55.7%, and 44.3% had tumors with an ER+, PR+, and HER2+ receptor status, respectively. A majority of the patients (n = 123) were diagnosed with invasive ductal carcinoma, and a smaller proportion (n = 26) with invasive lobular carcinoma. The patients had an average initial tumor sizes of 46.4 ± 27.1 mm. Pathologic assessment after surgery demonstrated 34% (n = 50) of patients achieved a pCR; whereas 66% (n = 99) were non-pCR. The patients in the training and test set had similar statistics in terms of clinical and pathological characteristics, and similar proportions of patients with pCR and non-pCR were randomly included in both sets.

**Table 1:** Demographic and clinical information of the patients involved in the study. The distribution of each variable was compared between the training and test sets using the Pearson's Chi-squared homogeneity test for categorical variables and the and *t* test for continuous variables; the p-values are reported in the last column.

Patient demographics and clinicopathologic characteristics	Count (%)				p-value
	Training (n = 111)		Test (n = 38)		
	pCR (n = 39)	Non-pCR (n = 72)	pCR (n = 11)	Non-pCR (n = 27)	p = 0.49
Median age (years)	48.4	52.2	50.6	52.1	p = 0.33
**Menopausal status**					
Pre/peri-menopausal	22 (56%)	36 (50%)	6 (55%)	15 (56%)	p = 0.75
Post-menopausal	17 (44%)	36 (50%)	5 (45%)	12 (44%)	
**Receptor status**					
ER positive	14 (36%)	53 (74%)	2 (18%)	18 (67%)	p = 0.27
PR positive	13 (33%)	43 (60%)	1 (9%)	16 (59%)	p = 0.11
HER2 positive	27 (69%)	22 (31%)	7 (64%)	10 (37%)	p = 0.32
**Histology**					
Invasive ductal carcinoma	39 (100%)	63 (88%)	11 (100%)	10 (37%)	p = 0.49
Invasive lobular carcinoma	0(0%)	9 (9%)	0 (0%)	17 (63%)	
**Nottingham grade**					
1	1 (2%)	3 (4%)	0 (0%)	0 (0%)	p = 0.50
2	9 (23%)	36 (50%)	2 (18%)	18 (67%)	
3	29 (75%)	33 (46%)	9 (82%)	9 (33%)	
**Tumor size**					
Mean tumor size (mm; ± SD)	37.2 ± 21.2	50.9 ± 28.9	44.1 ± 25.8	48.9 ± 27.9	p = 0.78
**Other clinical information**					
Inflammatory breast cancer	4 (10%)	10 (14%)	0 (0%)	1 (4%)	p = 0.53

**Figure 2:**
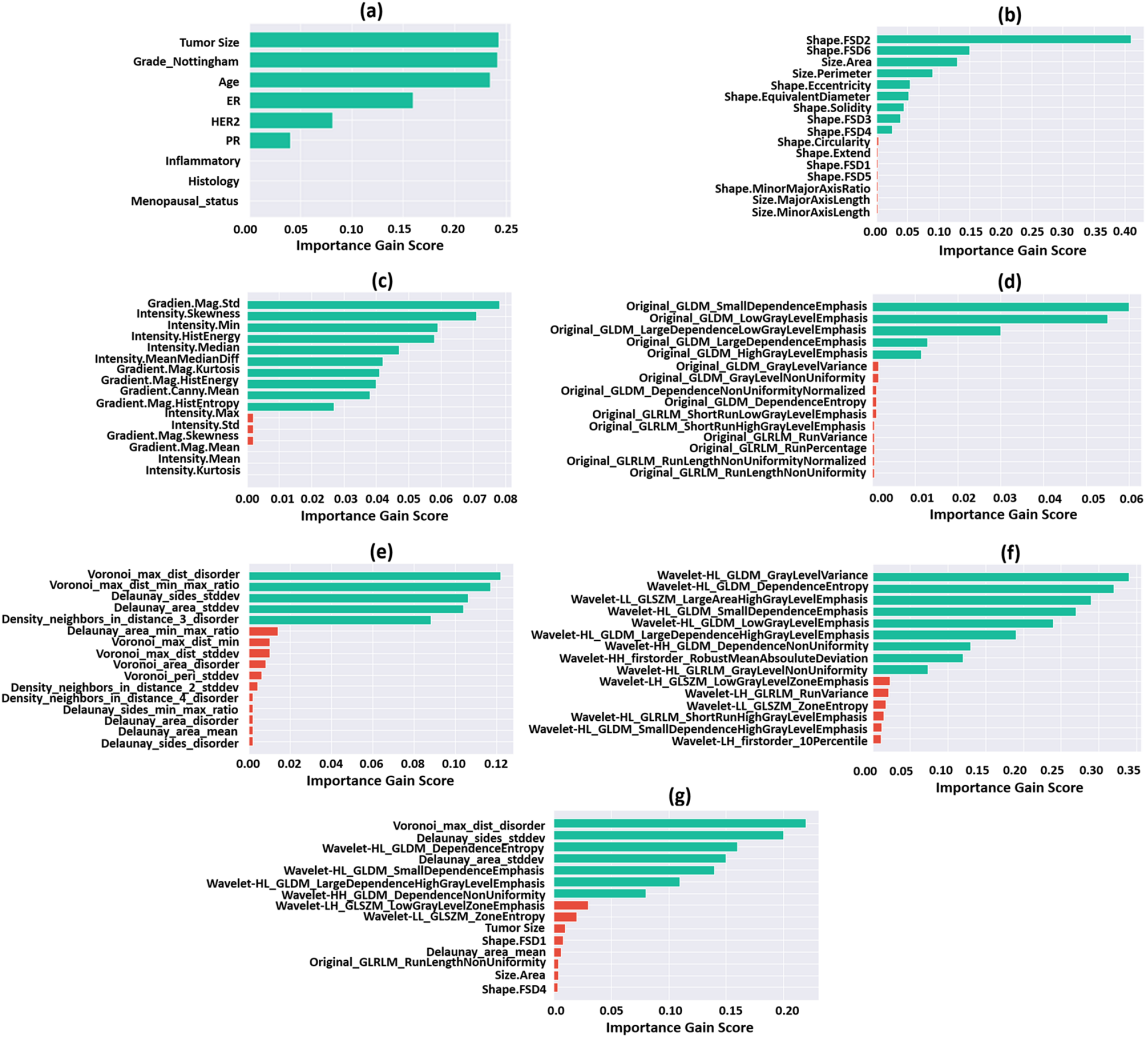
The importance gain score of the first 15 features with highest contribution to the predictive model for different feature subsets: (**a**) clinical, (**b**) morphological, (**c**) intensity-based, (**d**) texture, (**e**) graph-based, (**f**) wavelet, and (**g**) all features. The green bars are associated with the features included in the NAC response biomarker in each experiment.

**Figure 3:**
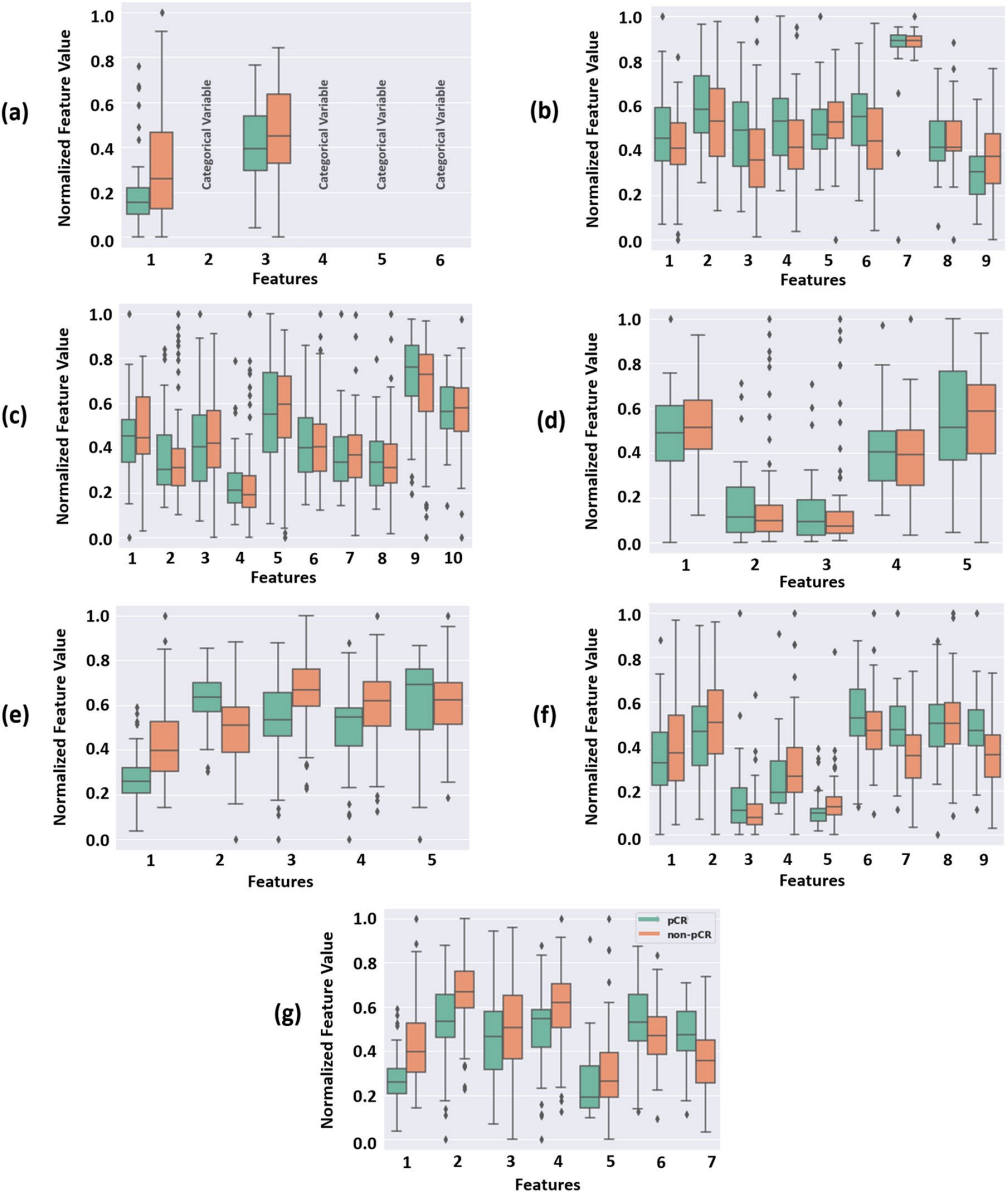
Box plots of the selected features for the pCR and non-pCR cohorts of the training set obtained in the seven experiments conducted using different feature subsets: (**a**) clinical, (**b**) morphological, (**c**) intensity-based, (**d**) texture, (**e**) graph-based, (**f**) wavelet, and (**g**) all features. The feature values are normalized in the range of 0 and 1. The order of features in each plot is the same as that of the associated plot in Fig. 2.

Figure 2 shows the importance gain score of the first 15 features with the highest contribution to each predictive model. The best features were selected in each experiment based on the importance gain score as shown in the figure. In the first experiment, six clinical features including the tumor size, Nottingham grade, age, as well as the ER, HER2, and PR status demonstrated a non-zero importance gain score and were selected for model development. In the second to sixth experiments, nine morphological features, ten intensity-based features, five texture features, five graph-based features, and nine wavelet features demonstrated a notable difference in their importance gain score compared to the rest of the features and were selected as the best features. Similarly, in the last (seventh) experiment that incorporated all the feature subsets (clinical and pathomic features), the first seven features were selected and included in the biomarker as their importance gain score demonstrated a considerable difference compared to rest of the features. The selected features in this biomarker only include the pathomic features, with four texture features derived from the wavelet-filtered images, and three graph-based features extracted from the tumor nuclei masks. Figure 3 demonstrate the box plot of the selected features in different experiments for the pCR and non-pCR populations of the training set. The plots in Fig. 3a–f shows a relatively good separation in statistical distribution of the selected features between the two groups, particularly for those in the graph-based and wavelet feature subsets. The features selected among all feature subsets in the last experiment (Fig. 3g) demonstrate a very good separation between the quartiles and median of feature values obtained for the two cohorts.

**Figure 4:**
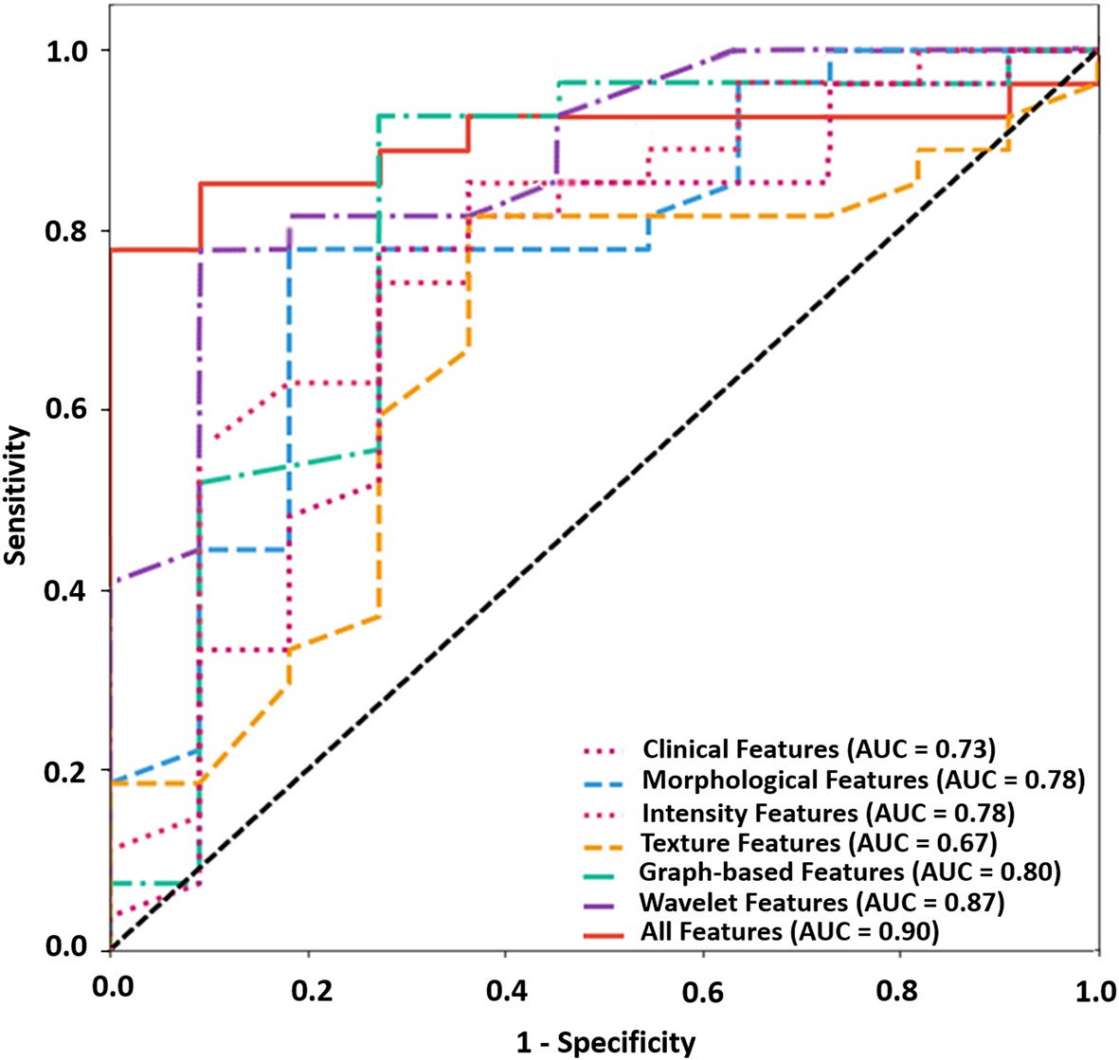
Receiver operating characteristic (ROC) curves on the independent test set for the predictive models developed with the selected features obtained in different experiments. In the last experiment and from all feature subsets, 7 wavelet and graph-based features were selected.

The evaluation results of the predictive models developed in different experiments have been presented in Table 2. The training and five-fold cross-validation accuracies of the developed models were very close together in each experiment and in the range of 72–85% and 71–84%, respectively. The perdition performance of the models on the independent test set has also been reported in the table. The test accuracy, sensitivity, and specificity of the models in different experiments were in the range of 71–84%, 70–85%, and 64–82%, respectively. The best results were obtained in the seventh experiment with the response biomarker consisting of the wavelet and graph-based features with a test accuracy of 84%, a sensitivity of 85%, and a specify of 82%. The ROC curves obtained on the independent test set is shown in Fig. 4 for different models. The AUC of the models ranged between 0.67 and 0.90 with the best result associated with the model developed using the wavelet and graph-based features.

**Table 2:** Results of NAC response prediction at pre-treatment using the clinicopathological and/or pathomic features, on the training, validation and test sets. The features included in each optimal biomarker have been listed in Fig. 2. For the validation set, the 95% confidence intervals are reported over the five folds of cross validation. The best value in each column is in bold.

Features	Number of features in optimal biomarker	Tr Acc	Val Acc	Val AUC	Val Sen	Val Spec	Te Acc	Te AUC	Te Sen	Te Spec
Clinical features	6	0.72	0.71 ± 0.02	0.74 ± 0.04	0.70 ± 0.04	0.73 ± 0.03	0.71	0.73	0.70	0.73
Morphological features	9	0.76	0.76 ± 0.03	0.78 ± 0.05	0.78 ± 0.03	0.67 ± 0.02	0.74	0.78	0.78	0.64
Intensity-based features	10	0.78	0.75 ± 0.02	0.77 ± 0.03	0.77 ± 0.02	0.69 ± 0.02	0.74	0.78	0.78	0.64
Texture features	5	0.76	0.75 ± 0.01	0.70 ± 0.03	0.78 ± 0.02	0.68 ± 0.03	0.74	0.67	0.78	0.64
Graph-based features	5	0.75	0.76 ± 0.01	0.79 ± 0.02	0.79 ± 0.03	0.72 ± 0.03	0.76	0.80	0.78	0.73
Wavelet features	9	0.82	0.83 ± 0.02	0.84 ± 0.03	0.80 ± 0.04	**0.83 ± 0.02**	0.82	0.87	0.81	**0.82**
All features	7	**0.85**	**0.84 ± 0.03**	**0.89 ± 0.04**	**0.84 ± 0.04**	0.82 ± 0.02	**0.84**	**0.90**	**0.85**	**0.82**

*Acc* accuracy, *AUC* area under the curve, *Sen* sensitivity, *Spec* specificity, *Tr* training, *Val* validation, *Te* test.

## Discussion

In this study, a GBM multi-feature ML model was investigated with various sets of clinicopathological and quantitative pathomic features derived from pre-treatment core biopsy specimens to predict the therapy response of BC patients undergoing NAC. Seven experiments were conducted to explore the efficacy of various feature subsets in predicting the therapy outcome. The results demonstrated a superior performance of the wavelet and graph-based feature in predictive modeling of NAC response at pre-treatment. The best results were obtained in the final experiment where all the clinical and pathomic features were included in the initial feature set. The response signature developed in this experiment consisted of seven features including four wavelet and three graph-based features. Results of descriptive analysis demonstrated a promising separation among the quartiles and median of these features between the two response cohorts. The ML model developed using this biomarker predicted the NAC response of the patients in the independent set with a sensitivity, specificity and AUC of 85%, 82% and 0.90, respectively. The multivariate GBM demonstrated that the non-linear combination of the selected pathomic features has a very good predictive ability for NAC response at pre-treatment.

The results of experiments conducted in this study demonstrated that the pathomic features could outperform the clinical variables in NAC response prediction. Whereas a few pathomic feature subsets including the morphological, intensity-based and texture features could differentiate the response cohorts with slightly better prediction accuracy compared to the clinical features, the wavelet and graph-based features demonstrated a considerably better efficacy. This observation was further confirmed in the last experiment where among all features, only the pathomic features from these two subsets were selected in the optimal biomarker with no feature from the clinicopathologic subset. Among the seven features included in this biomarker, the three graph-based features characterize the variations in spatial distribution of the intra-tumor nuclei with different measures. Specifically, the Voronoi_Max_Distance_Disorder feature measures the variations in maximum distance within polygons in Voronoi diagram of the nuclei, while Delaunay_Sides_Stddev and Delaunay_Area_Stddev measure the variations in side length and area of triangles in Delaunay triangulation graph generated using the Voronoi partitions^35^. The wavelet features selected in the biomarker, on the other hand, characterize the spatial heterogeneity within the tumor nuclei by quantifying the gray-level dependencies in the associated wavelet-filtered images. Specifically, the Wavelet_HL_GLDM_DE, Wavelet_HL_GLDM_SDE, Wavelet_HL_GLDM_LDHGLE, Wavelet_HH_GLDM_DNU features measure the entropy in gray-level intensity dependence, texture homogeneity, distribution of close similarities with higher intensity values, and the uniformity of intensity values within the nuclei^36^.

Imaging features confer information about cell–cell interactions and activity within the tumor microenvironment^37^. Determining actionable biomarker signatures, derived by mapping tumor subcomponents and characterizing the biological heterogeneity has the potential to improve diagnosis and response-guided treatment strategies. Previous studies have investigated the efficacy of the pathomic features in conjunction with the genomic features for other applications of cancer diagnosis and prognosis^38,39^. The findings of those studies are in agreement with the observations in this study. The genomic data, however, are not routinely acquired for LABC. Therefore, incorporating these parameters in predictive modeling of therapy outcome requires extra data acquisition and processing that may not be always feasible. A number of other studies have explored the performance of quantitative imaging data (radiomic features) acquired at early stage of diagnosis for NAC response prediction^40–42^. The observations of those studies confirm that the BC characteristics such as intra-tumor heterogeneity quantified using pretreatment imaging can reasonably be correlated to the NAC outcome. One limitation associated with predictive modeling using the imaging-based features is that the performance of such systems could possibly be affected by imaging acquisition protocols including variations in resolution, magnification, and gain parameters^43^. A number of previous studies have focused on post-treatment nonsurgical techniques including biopsy and imaging to detect residual cancer in the breast or axilla after NAC^44,45^. Specifically, pre-surgical vacuum-assisted biopsy (VAB) coupled with machine learning methods have been investigated to identify patients with pCR to NAC who may not need to undergo surgery. The results demonstrate that combining the clinical, imaging and VAB variables integrated with machine learning models can improve the performance in pre-surgical NAC response identification. Whereas applying such methods at post-treatment may spare the patients with pCR from an unnecessary mastectomy or lumpectomy, they cannot facilitate treatment adjustments or switching to alternative treatments for non-responders.

The results of this study were obtained using a relatively small dataset (n = 149) acquired from a single institution. A test set was randomly selected and kept completely unseen during the model development and tuning to assess the performance of the models independently. Whereas similar performance of the models on the validation and independent test sets can imply a good generalizability of the models on unseen samples, no external test set was available in this study to minimize the chance of bias in model evaluations. As such, to evaluate the robustness of the methods and assess the performance and applicability of the developed models in clinic rigorously, further investigations are required on larger cohorts of patients with multi-institutional data.

In conclusion, this study demonstrated a very good potential of hand-crafted pathomic features integrated with ML techniques in predicting the pathological response of BC patient to NAC. The promising results obtained in this study is a step forward towards a priori chemotherapy response prediction in high-risk BC patients using smart quantitative histopathology methodologies at pre-treatment. Early prediction of NAC response permits timely therapy adjustment by oncologists or switching to more effective treatment for individual patients. A personalized oncology paradigm for BC patients is expected to improve their overall therapy outcome and quality of life. The promising results obtained in this study pave the way for further investigations and encourage future studies to integrate more advanced ML methodologies including the end-to-end deep learning architectures with digital histopathology for NAC response prediction.

## Data Availability

Data were collected and available at the Odette Cancer Centre, Sunnybrook Health Sciences Centre, Toronto, ON, Canada.
